# Spinning Disk Confocal Microscopy for Optimized and Quantified Live Imaging of 3D Mitochondrial Network

**DOI:** 10.3390/ijms25094819

**Published:** 2024-04-28

**Authors:** Somaieh Ahmadian, Patrick J. Lindsey, Hubert J. M. Smeets, Florence H. J. van Tienen, Marc A. M. J. van Zandvoort

**Affiliations:** 1 Department of Toxicogenomics, Maastricht University Medical Centre+, 6229 ER Maastricht, The Netherlands; patrick.lindsey@maastrichtuniversity.nl (P.J.L.); bert.smeets@maastrichtuniversity.nl (H.J.M.S.); florence.vantienen@maastrichtuniversity.nl (F.H.J.v.T.); 2 GROW Research Institute for Oncology and Reproduction, Maastricht University, 6229 ER Maastricht, The Netherlands; 3 Department of Genetics and Molecular Cell Biology, Maastricht University, 6229 ER Maastricht, The Netherlands; 4 Institutefor Mental Health and Neurosciences (MHeNS), Maastricht University Medical Centre+, 6229 ER Maastricht, The Netherlands; 5 Cardiovascular Research Institute Maastricht (CARIM), Maastricht University, 6229 ER Maastricht, The Netherlands; 6 IMCAR, Institute for Molecular Cardiovascular Research, Universitätsklinikum Aachen, 52074 Aachen, Germany

**Keywords:** JC-1, MitoTracker Red CMX Rox, TMRM, CLSM, spinning disk confocal microscope, mitochondria

## Abstract

Mitochondria are the energy factories of a cell, and depending on the metabolic requirements, the mitochondrial morphology, quantity, and membrane potential in a cell change. These changes are frequently assessed using commercially available probes. In this study, we tested the suitability of three commercially available probes—namely 5′,6,6′-tetrachloro-1,1′,3,3′-tetraethylbenzimidazolo-carbocyanine iodide (JC-1), MitoTracker Red CMX Rox (CMXRos), and tetramethylrhodamine methyl ester (TMRM)—for assessing the mitochondrial quantity, morphology, and membrane potential in living human mesoangioblasts in 3D with confocal laser scanning microscope (CLSM) and scanning disk confocal microscope (SDCM). Using CLSM, JC-1, and CMXRos—but not TMRM—uncovered considerable background and variation. Using SDCM, the background signal only remained apparent for the JC-1 monomer. Repetitive imaging of CMXRos and JC-1—but not TMRM—demonstrated a 1.5–2-fold variation in signal intensity between cells using CLSM. The use of SDCM drastically reduced this variation. The slope of the relative signal intensity upon repetitive imaging using CLSM was lowest for TMRM (−0.03) and highest for CMXRos (0.16). Upon repetitive imaging using SDCM, the slope varied from 0 (CMXRos) to a maximum of −0.27 (JC-1 C1). Conclusively, our data show that TMRM staining outperformed JC-1 and CMXRos dyes in a (repetitive) 3D analysis of the entire mitochondrial quantity, morphology, and membrane potential in living cells.

## 1. Introduction

Mitochondria are important organelles in most eukaryotic cells, and they are mainly known for producing energy in the form of ATP via the oxidative phosphorylation system in the mitochondrial inner membrane. Additionally, mitochondria have other roles, including Ca^2+^ homeostasis, which is important for cell fate, e.g., for proceeding or inhibiting apoptosis [1]. The mitochondrial membrane potential, together with the mitochondrial proton gradient, is not only important for ATP production but also for mitochondrial calcium influx [2]. Depending on the metabolic requirements of a cell, the mitochondrial morphology, quantity, and distribution in a cell change [3]. For example, pluripotent stem cells rely on glycolysis for ATP production, and their mitochondrial morphology is reported as a globular structure [4]. In contrast, high-energy-requiring differentiated cells rely more on oxidative phosphorylation and have a more tubular mitochondrial structure, which provides a larger surface for oxidative phosphorylation to take place [5,6,7]. It has been suggested that the mitochondria-to-cytoplasm ratio increases during cell differentiation [8]; therefore, the information on 3D mitochondrial morphology and membrane potential will be vital for mitochondrial function and state. One option for assessing the mitochondrial ultra-structure in a cell is electron microscopy, which provides very detailed information on the mitochondrial ultra-structure. However, electron microscopy does not provide any information regarding the mitochondrial membrane potential, nor can it be used for live-cell imaging. Moreover, in order to study the overall 3D mitochondrial network of large numbers of cells, electron microscopy requires expertise processing and is time-consuming [9]. In contrast, confocal microscopy does not only allow for assessing the mitochondrial ultra-structure, but it has sufficient resolution for studying the 3D mitochondrial network in live cells, with the possibility of assessing the mitochondrial membrane potential. Confocal laser scanning microscope (CLSM) uses a scanner to image the information through consecutive pinholes one by one. In contrast, in a Nipkow/Yokogawa confocal scanning unit of a spinning disk confocal microscope (SDCM), thousands of pinholes are arranged spiral-wise and provide simultaneous information on multiple points in the specimen [10]. While this is achieved at the cost of resolution, one gains imaging speed [11]. As CSLM and SDCM are commonly available in many institutes, we assessed both in this study.

For the confocal microscopy analysis of mitochondrial morphology and membrane potential in living cells, multiple fluorescent probes are available; the ones frequently used are 5,5′,6,6′-tetrachloro-1,1′,3,3′-tetraethylbenzimidazolocarbocyanine iodide (JC-1), MitoTracker Red CMX Rox (CMXRos) [12], and tetramethylrhodamine methyl ester (TMRM) [13,14,15]. JC-1—a cationic fluorescent dye—accumulates in the mitochondrial membrane as a monomer emitting green fluorescence. Additionally, red fluorescence is emitted by the aggregates of JC-1, which accumulate inside the mitochondria with a higher membrane potential. The formation of the aggregates is dependent on the electrochemical gradient; therefore, JC-1 allows for ratio-metric studies of the membrane potential in the mitochondria (ΔΨ*m*) [16]. Therefore, JC-1 can provide both structural and functional information on the mitochondria in living cells. CMXRos accumulates into the mitochondria in response to the highly negative mitochondrial membrane potential. After entering the mitochondria, each CMXRos forms a covalent bond with the thiol group of peptides and proteins via their reactive chloromethyl group. Consequently, CMXRos retains its signal even after the loss of the mitochondrial membrane potential—for example, after fixation [17,18]—and is thus suitable for imaging the structure of the mitochondrial network but not for assessing changes in the membrane potential in living cells. Like JC-1, TMRM is also a cationic cell-permeable dye used to visualize the mitochondria and accumulates in the mitochondrial membrane. The fluorescent signal of TMRM is proportional to the concentration of the probe, in turn increasing with mitochondrial membrane potential [19]. Therefore, while TMRM can be used to assess the membrane potential in a proportional manner only, JC-1 can quantify the membrane potential in a ratio-metric manner. 

The aim of this study was to determine the optimal dye (JC-1, CMXRos, or TMRM), imaging setup, and microscope (CLSM or SDCM) for 3D mitochondrial imaging in two types of living cells, namely mesoangioblasts and mesoangioblasts differentiated into myotubes. Unlike CMXRos and JC-1, TMRM has very low retention after fixation; therefore, the aim of the study was to find a protocol for live imaging of the cells. In combination with a modified script, this enables semi-automatic quantification of the changes in mitochondrial morphology and membrane potential, as exemplified using mesoangioblasts and mesoangioblast-derived myotubes.

## 2. Results

### 2.1. CLSM Imaging of Mitochondrial Network in Mesoangioblasts Stained with JC-1, CMXRos, or TMRM

**Figure 1:**
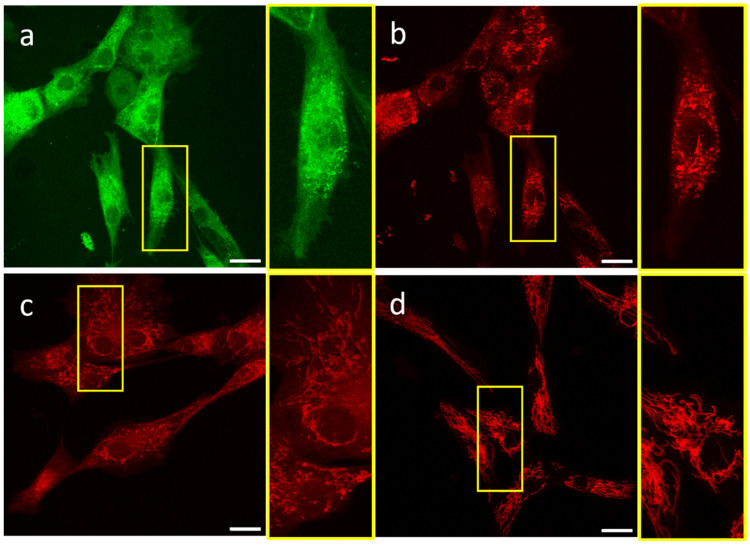
Representative images of mesoangioblasts stained with JC-1, CMXRos, and TMRM and imaged using CLSM. (**a**) JC-1 monomer (excitation: 488; emission: 510 ± 15 nm) representing depolarized mitochondria ((**b**–**d**): excitation: 560; emission: 579 ± 10 nm); (**b**) JC-1 aggregates representing polarized mitochondria; (**c**) CMXRos; (**d**) TMRM. Scale bars represent 20 μm. Each image is a maximum intensity projection for 70 stacks.

First, we carried out 3D imaging with CLSM using JC-1, CMXRos, or TMRM staining. We investigated whether these probes are suitable for studying the mitochondrial network in 3D in living human mesoangioblasts. Axial stacks of images of the cells were produced. As shown in Figure 1a, JC-1 monomer signals show many depolarized mitochondria but also a very strong, variable, and diffusive background. The signals from aggregated JC-1 (Figure 1b), representative of hyperpolarized mitochondria, are much sharper, but still, a considerable background is visible. With CMXRos, a considerable background is also present in the cytoplasm and nucleus (Figure 1c). In contrast, the mitochondrial network and morphology are clearly visible using TMRM, without significant background signals (Figure 1d).

### 2.2. SDCM Imaging of Mitochondrial Network in Mesoangioblasts Stained with JC-1, CMXRos, or TMRM

**Figure 2:**
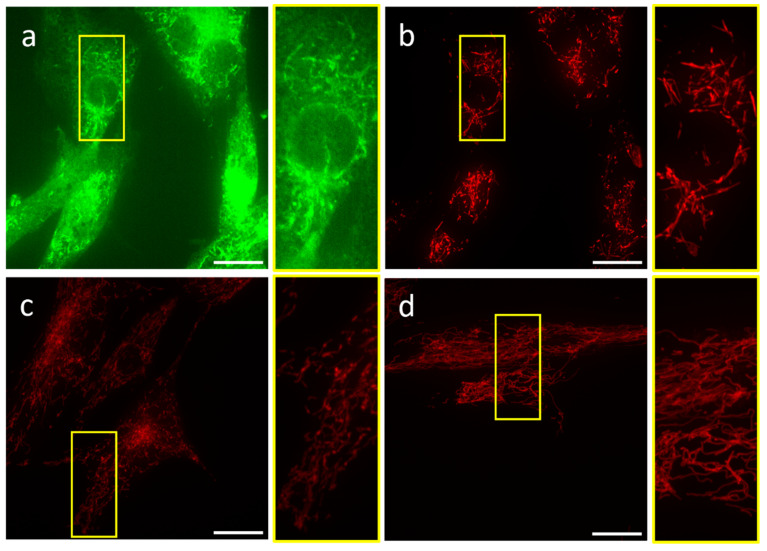
Representative images of mesoangioblasts stained with JC-1, CMXRos, and TMRM and imaged using SDCM. (**a**) JC-1 monomer (excitation: 488; emission: 514 ± 15 nm) representing depolarized mitochondria ((**b**–**d**): excitation: 561; emission: 590 ± 10 nm); (**b**) JC-1 aggregates representing polarized mitochondria; (**c**) CMXRos; (**d**) TMRM. Scale bars represent 20 μm. Each image is a maximum intensity projection for 70 stacks.

As SDCM provides faster image acquisition and lower laser exposure of the specimen, we investigated 3D imaging with SDCM using the three probes mentioned (Figure 2). For the green channel of JC-1, we observed considerable variable and diffusive background, as seen with CLSM (Figure 2a). In contrast, the background was negligible for the red channel of JC-1, and for both CMXRos and TMRM (Figure 2b–d). 

### 2.3. Consistency of Mitochondrial Morphology and Intensity upon Repetitive Imaging of JC-1, CMXRos, or TMRM Using CLSM and SDCM

**Figure 3:**
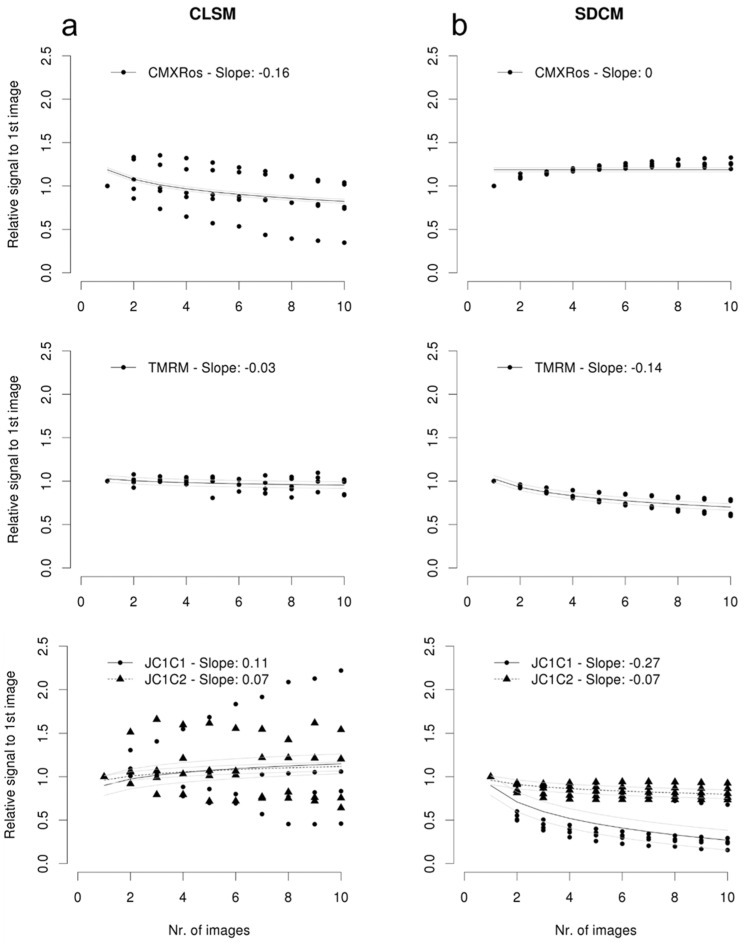

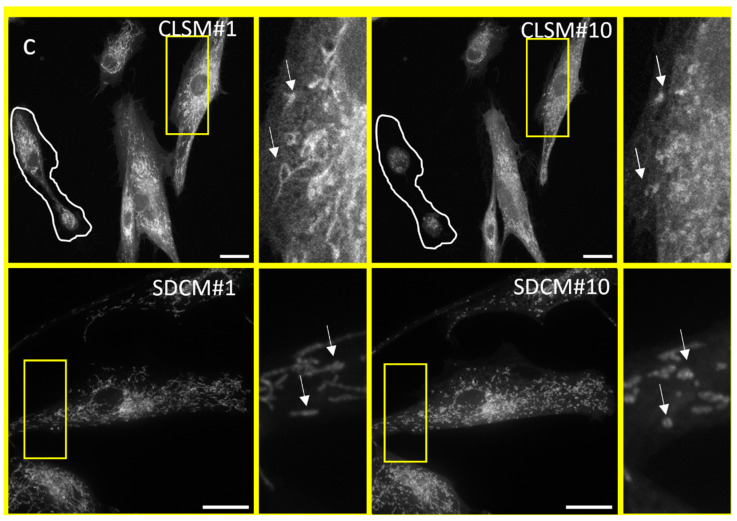
Quantification of TMRM, JC1, and CMXRos dye intensity changes, and morphological changes upon repetitive CLSM and SDCM imaging using CMXRos dye. (**a**,**b**) Average fluorescent intensity (black line) and standard error of the mean (gray lines) of 3–5 randomly selected ROIs (each datapoint is indicated with filled circle/triangle) in mesoangioblasts stained with either JC-1, CMXRos, or TMRM and imaged in 3D 10 times using CLSM (**a**) and SDCM (**b**), respectively. Note that the best fitting model for the CMXRos using SDCM is a horizontal line. JC-1 C1 and JC-1 C2 represent the red and green channel for JC-1, respectively; (**c**) Changes in mitochondrial morphology upon repetitive imaging of mesoangioblasts stained with CMXRos: Mitochondria in mesoangioblasts were stained with CMXRos, and 10 consecutive images were captured using CLSM and SDCM. Each image is a maximum intensity projection for 70 stacks. The microscope used (CLSM or SDCM) and the first or tenth image (#1 or #10, respectively) are indicated in each figure. Scale bars represent 20 μm. Mitochondrial morphology changes are indicated with a white outline and with arrows in the highlighted region.

In order to enable repetitive imaging, e.g., before and after the treatment of cells with a compound, we next assessed which combination of probe and microscopy techniques enabled consistent and reproducible mitochondrial morphology quantification. Mesoangioblasts were first stained with one of the three mentioned probes; then, 10 consecutive 3D stacks of 3–5 fields per sample were performed using CLSM and SDCM immediately after each other. The relative intensity of the mean fluorescent intensity in the maximum intensity projection images was quantified in Figure 3a,b. With CMXRos, a reduction (slope −0.16) was observed with CLSM imaging, while a stable signal was observed using SDCM imaging (slope 0). However, upon repetitive CLSM imaging of CMXRos, additional changes were observed both in the mitochondrial network and changes from rod-shaped to more globular mitochondria between the first and tenth stack from the same field of view (Figure 3c). With SDCM, no apparent changes in the mitochondrial network were observed, but changes in the mitochondrial morphology remained. Furthermore, as imaging with CLSM took much longer than with SDCM, we observed the movement of cells—and consequently, also mitochondria—during image acquisition, which might be the reason for the high standard deviation in the quantification of fluorescent intensity. Upon repetitive imaging of JC-1 using CLSM, a slight and nearly equal increase in the signal was observed for both channels (slope JC-1 C1 and JC-1 C2, respectively, 0.07 and 0.11), but a large variation between the cells was observed. The variation was less pronounced with SDCM imaging of JC-1 but demonstrated an unequal reduction in the signal between the two channels upon repetitive imaging (slope JC-1 C1 and JC-1 C2, respectively, −0.27 and −0.07). Importantly, CLSM imaging of TMRM showed minimal changes in signal intensity (slope −0.03) and less variation than CLSM imaging of the two former probes. Occasionally, mitochondrial network changes were observed between the first and tenth stack from the same field of view with CLSM imaging, which were not apparent with SDCM imaging (Supplementary Figure S1). With SDCM imaging of TMRM, a decrease in signal intensity (slope −0.14) was observed, but the variation between image 1 and 10 was still equal to the variation observed for CLSM. Based on these data—and since TMRM images were free of background, and no morphological changes to the cells or mitochondria were observed—we continued with SDCM imaging of TMRM stained cells for quantification of the mitochondrial network and membrane potential. 

### 2.4. Quantification of Mitochondria Stained with TMRM and Imaged Using SDCM in Mesoangioblasts and Myotubes

**Figure 4:**
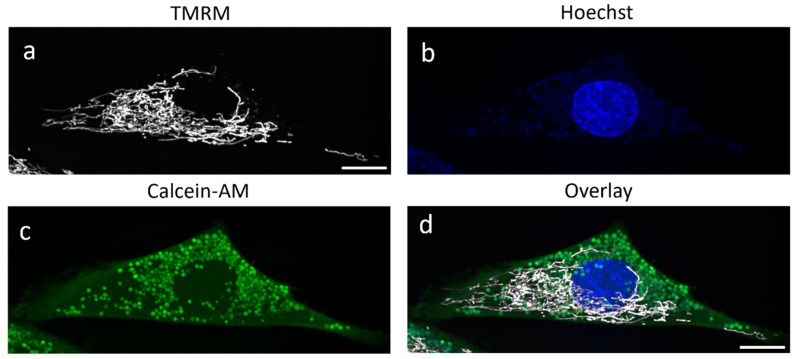
Maximum intensity projection of images of mitochondria, nucleus, and total cell area of mesoangioblasts using SDCM. TMRM staining (**a**); Hoechst staining of nucleus (**b**); Calcein-AM staining of cytoplasm (**c**); and overlay (**d**) of mitochondria in a mesoangioblast imaged in 3D using SDCM. Scale bar represents 10 μm.

The imaging of TMRM with CLSM demonstrated stable signal upon repetitive imaging, but imaging using SDCM was preferred because it was faster and because no changes in the mitochondrial morphology were observed. For a quantitative analysis of the mitochondrial network in SDCM-generated images, a script from the work of Iannetti et al. (see Section 4) [20] was adapted to analyze the 3D volumes. Mesoangioblasts were stained with TMRM to visualize the mitochondrial network; Hoechst 34580 to visualize the nucleus; and Calcein-AM to visualize the whole cell (see Section 4) (Figure 4).

**Figure 5:**
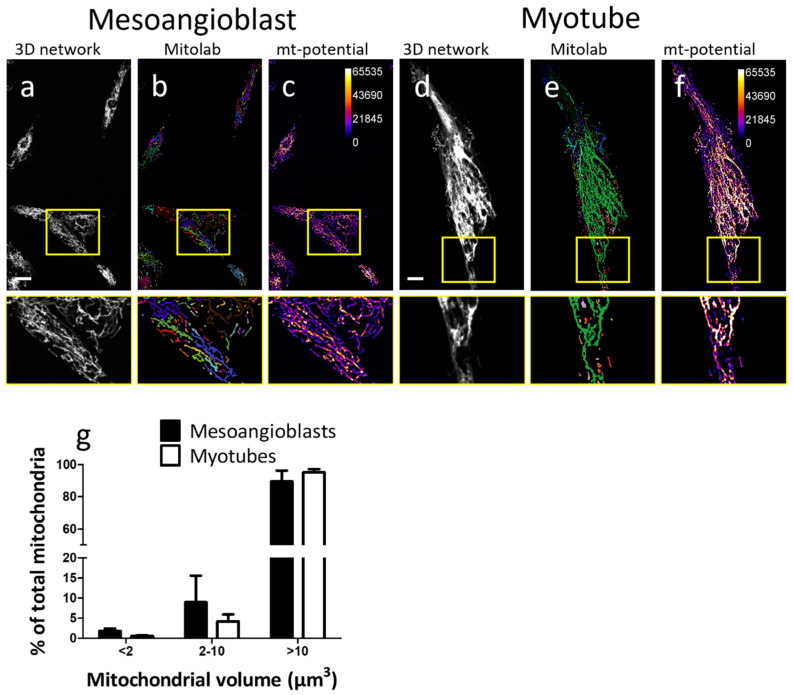
Mitochondrial quantification in mesoangioblasts and myotubes. (**a**–**c**) Mesoangioblast and (**d**–**f**) Myotube. (**a**,**d**) Mitochondrial network stained with TMRM and imaged using SDCM in mesoangioblasts and myotube, respectively; (**b**,**e**) Mitochondrial segmentation, with each color representing a separate mitochondrial object, which is not connected to the rest of the mitochondrial network; and (**c**,**f**) Qualitative image of maximum intensity projection of TMRM probe, representative of mitochondrial membrane potential, with the brighter area having higher membrane potential; (**g**) Percentage of mitochondrial objects per mesoangioblast/myotube, which have a volume of <2, 2–10, or >10 µm^3^. Scale bars represent 10 μm. Quantification is performed for 10 mesoangioblasts and 10 myotubes.

As an example, ten mesoangioblasts and ten mesoangioblast-derived myotubes were analyzed (one mesoangioblast/myotube per image) and quantified. Figure 5a,d show the maximum intensity projection of mitochondrial networks in mesoangioblasts and a myotube, respectively. Figure 5b,e show the segmentation created with the script used to label mitochondrial objects, which were not connected to each other (MitoLab). Each mitochondrial object, i.e., connected mitochondria, is visible as one color. The volume and mean intensity of each of these mitochondrial objects and the number of mitochondrial objects per cell were quantified (Table 1; Supplementary Files S2 and S3). Finally, Figure 5c,f show the intensity of the TMRM signal, representative of the mitochondrial membrane potential per mitochondrial object. As an example, undifferentiated mesoangioblasts and mesoangioblasts differentiated into multi-nucleated myotubes were analyzed. As shown in Table 1, the total mitochondrial volume per cell (sum volume of all mitochondrial objects), the number of mitochondrial objects per cell, and cytoplasm volume are all increased upon differentiation of mesoangioblasts. In addition, the intensity of the mitochondrial membrane potential per myotube is 17-fold higher than that of undifferentiated mesoangioblasts. Of note, the signal intensity of the mitochondrial membrane potential can only be compared within one experiment and not between experiments, as performed in this example analysis. To enable an assessment of the changes in mitochondrial morphology, we classified the mitochondrial objects in the mesoangioblasts and myotubes into three groups: (1) smaller than 2 µm^3^, representing tiny fragmented mitochondria; (2) 2–10 µm^3^, representing medium mitochondria; and (3) larger than 10 µm^3^, representing mitochondrial networks, as proposed in a previous study [21]. Figure 5 exemplifies how TMRM staining followed by 3D SDCM imaging can be applied to assess the changes in the mitochondrial network upon differentiation. 

### 2.5. TMRM Staining and Imaging Using SDCM Can Be Applicable to Other Cell Types

Following the analysis of mesoangioblasts and myotubes, we investigated whether TMRM imaging with SDCM can be used for other cell types. Therefore, we imaged undifferentiated hESCs, which are very small cells with a compact mitochondrial network, and fibroblasts, which are larger than mesoangioblasts but smaller than myotubes. We applied the same probe concentration and imaging protocol as the one used for the mesoangioblasts and described in Section 4. We noticed that this proposed imaging method for visualization of the mitochondrial network indeed provides bright staining without a background and without morphological changes in the mitochondrial network. Examples of mitochondrial network visualization for fibroblasts and human embryonic stem cells are shown in Supplementary Figure S2.

## 3. Discussion

The quantification of the full 3D mitochondrial network in living cells enables us to study the changes in mitochondrial volume, fission and fusion, and/or mitochondrial membrane potential due to, e.g., cell differentiation, interventions, or genetic defects. The fluorescent microscope can be a valuable tool for 2D quantification of mitochondrial morphology in flat cells, such as primary human skin fibroblasts, with an axial dimension of 3 µm [22]. However, a 3D approach is required for analyzing the morphology of a dense mitochondrial network, which exists in larger differentiated cells, such as myotubes [23,24]. In order to quantitatively study the complete mitochondrial network in living cells in 3D, selecting a probe and imaging technique enabling clear visualization of that network without inducing changes is essential. To this end, we tested three frequently used probes—namely JC-1, CMXRos, and TMRM [25,26,27,28]—and used both CLSM and SDCM for 3D live-cell imaging and analysis. We observed that the background signals were lowest when using TMRM and that TMRM showed less variation in fluorescent intensity upon consecutive imaging when comparing the other two probes with both microscopes. Signal intensity reduction was slightly stronger with SDCM (slope −0.14) than with CLSM (slope −0.03) imaging of TMRM, but we did not observe mitochondrial network changes upon consecutive imaging with SDCM compared to an occasional change using CLSM. Additionally, in some images, we observed higher background signal with CLSM; this could be due to the high spot illumination of CLSM as compared to SDCM, which diffused the probes in the media [29]. In addition, phototoxicity, heating, photobleaching, and photodamage might be some of the factors influencing the changes we observed. However, as we kept all the influencing factors—such as staining and image acquisition—equal for all probes, we conclude that TMRM staining can be performed with both imaging setups but that TMRM staining in combination with SDCM imaging is the most suitable out of the three tested probes for 3D live-cell imaging. In line with our data, for which 200 nM TMRM was used, TMRM is reported to be more frequently used due to having less toxicity—below 250 nM—and less photobleaching/phototoxicity [30].

JC-1 requires a ratio-metric determination of its fluorescence in both green and red wavelength regions. Like TMRM, the aggregated JC-1 signal (red channel) also showed clear mitochondrial networks, but with considerable background, while the green signal from monomeric JC-1 appeared to be diffusively distributed in cells, especially when using CLSM. Repetitive imaging of JC-1 using CLSM showed increasing variation in signal intensity between the cells, namely up to 2.2-fold for JC-1 C1 and 1.5-fold for JC-1 C2. In contrast, repetitive SDCM imaging showed less variation in signal between the cells but demonstrated unequal decay (JC-1 C1 slope of −0.027 compared to JC-1 C2 slope of −0.07), which would greatly impact the ratio-metric quantification. Moreover, the required use of two wavelength regions for JC-1 quantification makes it difficult to combine JC-1 with other fluorescent dyes in the green–red range, such as the Calcein-AM probe for measuring cell volume. Additionally, it has been reported that the fluorescence of JC-1 was affected by variables such as antioxidants and resulted in a decrease in the membrane potential, which was not observed with TMRM [27,28,31]. As observed for the JC-1 aggregates, we also noticed background signals with CMXRos, specifically using CLSM. Repetitive imaging using CLSM of CMXRos stained cells also showed a 1.5-fold variation in signal intensity between the cells and a reduction of −0.14 in slope. Repetitive imaging using SDCM resolved signal intensity variation and showed no decay in signal intensity; however, the apparent changes in mitochondrial morphology remained. MitoTrackers, including CMXRos, establish covalent bonds with the thiol group of proteins and peptides, making them ideal for quantification of mitochondrial volume, as the signal is supposed to be unchanged even if the mitochondrial membrane potential is lost due to the covalent band. However, this makes them unsuitable for repetitive measurements of the same cells in order to assess the effects of an intervention, since the intervention will not change the mitochondrial signal intensity. Their signal may also be strongly influenced by the presence of reactive oxygen species and impair respiration, as reported by Buckman et al. [25].

We would like to emphasize that our conclusion of using TMRM staining in combination with SDCM imaging is based on our image acquisition details. However, one could reduce the damage to mitochondria by using other imaging settings for CLSM, reducing the scanning time (e.g., using a resonant scanner), or setting lower laser power and line accumulation combinations. However, low 3D speed and limited sensitivity might be the limiting factors in performing 3D imaging of the mitochondrial network of an entire cell. Alternatively, ProLong™ Live Antifade Reagent can be added to the medium. As this reagent is proposed to function as an antioxidant reagent, and we wanted to avoid any confounding factors for mitochondrial functioning, we decided to not use it and focus on a faster and less damaging 3D imaging method. SDCM indeed provides faster image acquisition but at the cost of resolution compared to CLSM. As a consequence, it is possible that we overestimated the size of mitochondrial objects if two or more mitochondrial objects were located closer to each other than the distance, which could be distinguished by resolution of the SDCM [32]. Moreover, SDCM suffers from several drawbacks. The most significant limitation of SDCM lies in the pinhole cross-talk effect, which means that emissions originating from distant focal planes and scattered out-of-focus light pass through the neighboring pinholes, resulting in a blurry background signal and affecting the axial resolution. This increases the background signal for thicker specimens [33]. However, in our scenario of cell imaging, this effect is not expected to play a significant role, since the cell cultures used are very thin. Another drawback is the limited level of light passing through the disk, which limits the imaging of faint fluorescent samples [34]. However, in our case, the fluorescence was quite strong for all probes. Additionally, SDCM lacks a scan zoom function, while the limitation of modifying the size of the pinhole restricts the manipulation of optical sectioning strength and imaging resolution [35]. Since we looked at the cell as a whole, this was not restrictive in our case. However, if all the settings for imaging and image processing are kept the same within one experiment for all probes, the SDCM-generated 3D images of TMRM stained cells can be used to assess the effect of treatment on the mitochondrial network and membrane potential, as demonstrated in multiple studies [21,22,36,37,38]. Another drawback of our study is that we only assessed the cationic probes for fast live imaging, and we did not compare how these probes are comparable with other mitochondrial markers—for example, antibody staining for specific mitochondrial proteins, where cell fixation is needed.

Following the identification of the combination of TMRM and SDCM as the best dye and imaging setup for live imaging of mesoangioblasts, a comprehensive script was adopted from the work of Iannetti et al. [20] to quantify in 3D the mitochondrial network, nucleus, and cytoplasm volume after obtaining the z-stack images for the entire network of mitochondria in cells with SDCM. This 3D quantification method for mitochondria is a relatively easy and fast method for analyzing the mitochondrial volume, network organization, and intensity in multiple images of multiple samples simultaneously. As mitochondrial changes are associated with myogenic differentiation [39], as an example, we quantified the total mitochondrial volume, intensity, and network of mesoangioblasts before and after differentiation into multi-nucleated myotubes (Table 1; Figure 5). 

Taken together, this study shows that TMRM staining outperforms JC-1 and CMXRos dyes in the (repetitive) analysis of entire mitochondrial networks and membrane potential in mesoangioblasts and myotubes without the need for ultra-structural details. TMRM 3D imaging is preferably performed with SDCM imaging, and the imaging data can be analyzed semi-automatically using the provided script. As the same imaging protocol for 3D mitochondrial visualization using TMRM for human embryonic stem cells and fibroblasts showed bright mitochondrial staining in the absence of background and changes in the mitochondrial network, we assume that 3D imaging protocols based on TMRM and SDCM might be applicable to a wide range of cell lines. We described and tested an imaging protocol, which could also be used for these cell lines.

## 4. Materials and Methods

### 4.1. Cell Culture and Staining of Cells

All materials were purchased from Thermo Fisher Scientific (Waltham, MA, USA) unless stated otherwise. The mesoangioblasts of controls were cultured according to a previously published study [40]. For mesoangioblasts’ passage, numbers 4–15 were used for all the experiments. For differentiation of mesoangioblasts into myotubes, mesoangioblasts were allowed to reach 100% confluence on Matrigel-coated 4-well microscopy µ slides (Ibidi); then, the medium was replaced with myogenic differentiation medium consisting of DMEM containing 2% horse serum. Myotubes were formed on day 10 after the start of differentiation. Dermal fibroblasts derived from healthy controls were cultured in complete medium containing DMEM, 10% FBS, and 1% pen/strep. Human embryonic stem cells (hESCs) were cultured on Matrigel-coated plates in Gibco’s Essential 8™ Flex media with supplement, according to the manufacturer’s protocol. All cells were cultured on 4-well microscopy µ slides. For mitochondrial analysis, cells were incubated with 2 μM JC-1, 100 nM CMXR, or 200 nM TMRM. For visualization of the cytoplasm, cells were incubated with 2 μM Calcein-AM, while for visualization of the nucleus, they were incubated with 2 μM Hoechst 34580. All staining was carried out in the medium for 30 min at 37 °C and with 5% CO_2_, based on the protocols provided by the manufacturers for each dye. After staining, fresh pre-warmed medium was added to the cells, and imaging was performed. 

### 4.2. Imaging of JC-1, CMXRos, and TMRM Using Confocal Laser Scanning Microscopy (CLSM)

Imaging of live cells was performed using a CLSM (Leica SP8, Leica Microsystems,, Amsterdam, The Netherlands) equipped with a white light laser (470–650 nm). JC-1 signals were obtained after excitation at 488 nm using a HyD detector (Leica) at 510 ± 15 nm for the monomers. For the aggregates of JC-1, for CMXRos, and for TMRM, excitation at 560 was applied, while emission was observed using a HyD detector (Leica Microsystems, Amsterdam, The Netherlands) at 590 ± 15 nm. The image acquisition details were as follows: scan speed = 400 Hz; pixel resolution (x, y) = 180 × 180 nm at 1024 × 1024 pixels per frame (leading to a frame acquisition of 2.56 s); number of z-slices = 70 with a z-distance = 140 nm; magnification of oil immersion objective = 63× with a NA = 1.4; pinhole size = 1 airy unit. 

### 4.3. Spinning Disk Confocal Microscopy (SDCM) Imaging of TMRM

Live cells were imaged with the CorrSight SDCM using Zeiss 63× oil immersion and 20× air-based objectives with numerical aperture (NA) of 1.4 and 0.8, respectively. The setup was equipped with an Andromeda spinning disk module (DSU; Olympus, Zoeterwoude, The Netherlands) and a Hamamatsu ORCA-Flash4.0 V2 camera (Hamamatsu Photonics, Hamamatsu City, Japan). Imaging was performed in the live mode using 4-well microscopy µ slides at 37 °C and 5% CO_2_. For imaging the mitochondria (JC-1 for the red channel, CMXRos, and TMRM), an excitation laser with a wavelength of 561 nm was used, while for the green channel of JC-1, an excitation laser with a wavelength of 488 nm was applied. Emission filters with wavelengths of FF01 593/46-25 and 514/30-25 nm were used, respectively. Mesoangioblasts were imaged using 63× oil objective for each probe. The image acquisition details were as follows: 63× oil immersion objective; pixel resolution (x, y) = 103 × 103 nm at 1656 × 1028 pixels; z-step size (70 frames) = 150 nm; frame exposure time = 50 ms.

For 3D quantitative acquisition of mitochondrial morphology and membrane potential in mesoangioblasts and myotubes, 20× air-based objective was used. Cells were stained only with TMRM, as described. For imaging the cytosol with Calcein-AM and the nuclei with Hoechst 34580, excitation lasers with wavelengths of 488 and 405 nm, respectively, and emission filters with wavelengths of FF01 523/30-25 and FF01 446 nm, respectively, were used. The final image acquisition details using the 20× objective were as follows: pixel resolution (x, y) = 325 × 325 nm at a frame consisting of 1656 × 1028 pixels. The z-step was calculated to be 350 nm, according to the optimal Nyquist sampling parameters. We imaged whole myotubes using 130 z-steps and whole mesoangioblasts using 70 z-steps. 

### 4.4. Quantification of Fluorescence Intensity upon Repetitive Imaging of JC-1, CMXRos, and TMRM Using CLSM and SDCM

Mesoangioblasts were stained with JC-1, CMXRos, or TMRM. For each dye, 10 consecutive volumes from the same field of view—each containing 70 images per stack—were generated using CLSM and SDCM. The image acquisition settings for each microscope regarding the zoom and laser power for the red channel of JC-1, CMXRos, and TMRM were kept the same for all probes. For each image, 3–5 different regions of interest (ROIs) with dimensions of 100–150 µm^2^ were selected and assessed using 10 maximum projection intensity images per dye and image setup, and the mean fluorescence intensity of each ROI was measured using Image J (2.14.0) > analyze > measure [41].

### 4.5. Quantitative Analysis of SDCM Images

Before quantification, deconvolution of 3D stacks was performed in Huygens Professional version 21.10 (SVI) using the classic maximum likelihood estimation algorithm, with a signal-to-noise ratio of 7.0, maximum iterations of 40, and quality threshold of 0.001. Deconvoluted images were segmented manually using maximum-intensity-projection Calcein-AM channel for the cell volume using ImageJ. Further calculations were performed using Matlab R2021a (The Mathworks, MA, USA.), with additional DipImage toolbox (TU Delft, The Netherlands), using a previously published script, which creates a mask of the raw data of each channel [20], which we adjusted to analyze multiple z-stacks. Using the script, deconvoluted images were first filtered both with 3D median and top hat filters and masked using thresholding at a constant value. In Calcein-AM images, holes, e.g., some vacuoles in a cell, were filled to calculate cell volume. The area of the Hoechst channel, representing the nucleus area, was quantified, as well as the Calcein-AM channel, which represents the whole cell volume. Nuclear volume was subtracted from whole cell volume to obtain cytoplasmic volume. Using the 3D mask, each mitochondrial object was labeled, and for each object, the size, intensity, and pixel size were calculated. The resulting output is an Excel file containing all the mitochondrial objects per cell, meaning all individual mitochondria, which are not connected to other mitochondria in the cytoplasm (Supplementary Files S2 and S3).

### 4.6. Statistical Analysis

In order to assess the effect of repetitive imaging, the relative signal of the first image was analyzed using a multivariate Gaussian non-linear regression for both the location and dispersion, including the time, dye, and type of microscope. The inference criterion used for comparing the models is their ability to predict the observed data, i.e., models are compared directly through their minimized minus log likelihood. When the numbers of parameters in the models differ, they are penalized by adding the number of estimated parameters—a form of Akaike information criterion (AIC) [42]. Thus, the AIC was used to assess which combination of dye and microscope had the smallest time trend and dispersion (i.e., which combination was the most stable over time). All statistical analyses presented were performed using the freely available program R v4.0.5 [43] and the publicly available library “gnlm” [44].

## Figures and Tables

**Table 1:** Example quantification results for mitochondrial network analysis of mesoangioblasts and myotubes.

	Number of mt Objects per Cell	Total mt Volume per Cell (*10^2^ µm^3^)	Cytoplasm Volume per Cell (*10^2^ µm^3^)	% mt Volume per Cytoplasm Volume	Total mt Intensity (*10^8^) per Cell ^#^
*Mesoangioblasts*	30 ± 12	7.14 ± 3.22	40.43 ± 14.98	17 ± 3.3	1.4 ± 0.7
*Myotubes*	167 ± 71	85.84 ± 41.51	294.02 ± 154.53	29.2 ± 14.1	24.3 ± 1.3

mt: mitochondrial. The data are presented as mean ± standard deviation. Quantification is performed for 10 mesoangioblasts and 10 myotubes. ^#^ Comparison of mt intensity is only possible for data generated from one staining and imaging session. In this example, the mesoangioblasts and myotubes were stained on different days, and therefore, the mt signal intensity cannot be compared.

## Data Availability

All data reported in this article are available in the article or in the Supplementary Materials.

## Supplementary Materials

The following supporting information can be downloaded at: https://www.mdpi.com/article/10.3390/ijms25094819/s1.
